# Tubulin cytoskeleton during microsporogenesis in the male-sterile genotype of *Allium sativum* and fertile *Allium ampeloprasum* L.

**DOI:** 10.1007/s00497-015-0268-0

**Published:** 2015-10-22

**Authors:** Dorota Tchórzewska, Kamil Deryło, Lidia Błaszczyk, Krystyna Winiarczyk

**Affiliations:** Department of Plant Anatomy and Cytology, Maria Curie-Skłodowska University, 20-033 Lublin, Poland; Department of Molecular Biology, Maria Curie-Skłodowska University, Lublin, Poland; Laboratory of Metabolomics, Institute of Plant Genetics of the Polish Academy of Sciences, Poznan, Poland

**Keywords:** *Allium sativum* L13, *Allium ampeloprasum* L., Microsporogenesis, Microtubular cytoskeleton, ITS sequences, Phylogenetic analysis

## Abstract

*****Key message***:**

**Microsporogenesis in garlic.**

**Abstract:**

The male-sterile *Allium sativum* (garlic) reproduces exclusively in the vegetative mode, and anthropogenic factors seem to be the cause of the loss of sexual reproduction capability. There are many different hypotheses concerning the causes of male sterility in *A. sativum*; however, the mechanisms underlying this phenomenon have not been comprehensively elucidated. Numerous attempts have been undertaken to understand the causes of male sterility, but the tubulin cytoskeleton in meiotically dividing cells during microsporogenesis has never been investigated in this species. Using sterile *A. sativum* genotype L13 and its fertile close relative *A. ampeloprasum* (leek), we have analysed the distribution of the tubulin cytoskeleton during microsporogenesis. We observed that during karyokinesis and cytokinesis, in both meiotic divisions I and II, the microtubular cytoskeleton in garlic L13 formed configurations that resembled tubulin arrangement typical of monocots. However, the tubulin cytoskeleton in garlic was distinctly poorer (composed of a few MT filaments) compared with that found in meiotically dividing cells in *A. ampeloprasum*. These differences did not affect the course of karyogenesis, chondriokinesis, and cytokinesis, which contributed to completion of microsporogenesis, but there was no further development of the male gametophyte. At the very beginning of the successive stage of development of fertile pollen grains, i.e. gametogenesis, there were disorders involving the absence of a normal cortical cytoskeleton and dramatically progressive degeneration of the cytoplasm in garlic. Therefore, we suggest that, due to disturbances in cortical cytoskeleton formation at the very beginning of gametogenesis, the intracellular transport governed by the cytoskeleton might be perturbed, leading to microspore decay in the male-sterile garlic genotype.

## Introduction

Commercially cultivated *Allium sativum* L. (garlic) is a completely sterile plant, incapable of sexual reproduction (Kamenetsky and Rabinowitch 2001; Shemesh Mayer et al. 2015). This agriculturally important plant reproduces exclusively in the vegetative mode, which is disadvantageous, because sexual reproduction in natural habitats ensures genetic variability contributing to evolutionary adaptation to changing environmental conditions (Gabrielsen and Brochmann 1998), but such breeding in commercial production may bring variability expected by consumers. Sterility in *A. sativum* is a secondary trait, which was evolutionarily conserved by vegetative reproduction. Anthropogenic factors seem to be the cause of the loss of sexual reproduction capability (Pooler and Simon 1993, 1994). The different genotypes within the *A. sativum* species were obtained by selection of spontaneous mutations rather than by sexual reproduction, which yielded plants that propagated only in the vegetative mode (Volk et al. 2004). In natural habitats, fertile *A. sativum* ecotypes grow only in the Tien Shan Mountains (on the border between Kazakhstan and China). Production of seeds and breeding these plants in different climate conditions failed, i.e. the capability of sexual reproduction disappeared gradually and resulted in full sterility (Etoh et al. 1988). Similarly, work aimed at restoration of fertility in garlic by selection of in vitro cultivated individuals failed. Although seed-producing individuals were obtained, they took a very long time to germinate (even 12 months), and the seedlings obtained exhibited morphological deformations (Jenderek 1998).

There are many different hypotheses concerning the causes of sterility in *A. sativum*. These include disturbances in the course of microsporogenesis, megasporogenesis, and gametogenesis (Kononkov 1953; Shemesh Mayer et al. 2013), abnormal development of the tapetum (Novak 1972), malnutrition of flowers resulting from competition between the generative and vegetative parts of the inflorescence (Koul and Gohil 1970; Winiarczyk 2009), degenerative-like diseases induced by mycoplasma or viruses (Konvicka 1973), and chromosomal deletions (Etoh 1986). The detailed investigations of the causes of garlic sterility conducted so far have comprised analyses of the environmental conditions and morphological distortions (Kamenetsky et al. 2003, 2004, 2005; Kamenetsky 2007; Shemesh Mayer et al. 2013). However, the mechanisms underlying male sterility in this species still cannot be comprehensively elucidated. Currently, the ongoing research is focused on elucidation of the causes and possibilities of restoring male fertility in garlic (Kamenetsky and Rabinowitch 2001, 2002; Kamenetsky et al. 2004; Bhagyalakshmi et al. 2005; Shemesh Mayer et al. 2013).

So far, the studies carried out on the microsporogenesis in *A. sativum* have revealed developmental disturbances at the different stages of the reduction division. The disturbances were manifested by changes in the nucleus ultrastructure (thinning of the content of the nuclei and appearance of light vesicles therein) and the presence of empty spaces in the cytoplasm, which indicated degeneration. In some anthers, degenerative processes were observed at the initial stage of meiosis (prophase), and this was the stage when anther degeneration took place. In other cases, in fact a majority of them, cytoplasm decay occurred at the final microsporogenesis stage—in microspore tetrads; no gametogenesis took place in such cells (Winiarczyk 2012). Until recently, no attention has been paid to the tubulin cytoskeleton in sterile genotype *A. sativum* meiotically dividing cells during microsporogenesis. In general, in plant cells, the tubulin cytoskeleton is an unusually labile structure, and any disturbances in the dynamically changing configurations of microtubules (MTs) result in disturbances of the entire microsporogenesis process. The formation of the functional male gametophyte has been described in maize (Liu et al. 1993), *Solanum* (Genualdo et al. 1998), and *Lavatera thuringiaca* (Tchórzewska et al. 2008). MTs are responsible for segregation of chromosomes and movement of organelles in a dividing cell during sporogenesis in monoplastidic hornworts (Brown and Lemmon 1990), homosporous fern (Giełwanowska et al. 2003), and microsporogenesis in higher plants (Tchórzewska et al. 2008). The cytoskeleton plays a major role in plant cytokinesis; for example, simultaneous cytokinesis described in *Lonicera japonica* and *Impatiens sultani* is strictly related to the tubulin cytoskeleton. In these species, the secondary spindles serve to position the four nuclei in the microsporocyte and the subsequently formed systems of microtubules radiating from the nuclei serve to define cleavage planes (Brown and Lemmon 1988). The tubulin cytoskeleton determines the plane of cell division during microsporogenesis, which has been perfectly documented in the irregular patterns of cytokinesis in orchids (Brown and Lemmon 1991). MTs are also responsible for vesicle transport and cellulose deposition (Hasezava and Nozaki 1999), thereby controlling plant morphogenesis (Gunning and Hardham 1982; Bulbert et al. 1998; Seagull 1990; Collings et al. 1999).

This paper describes comparative study of the tubulin cytoskeleton distribution during microsporogenesis in male-sterile *A. sativum* L13 (garlic) and fertile *A. ampeloprasum* L. (leek), a species that is closely related to garlic. Unlike garlic, leek reproduces sexually and produces numerous seeds; therefore, it is a suitable material to compare with the male-sterile garlic. The data presented in this paper describe the development of the microspore from the meiotic prophase to mononuclear microspore stage in both plants. The investigations show part of the complex microsporogenesis process with the aim of elucidation of the causes of male sterility in garlic.

## Materials and methods

### Plant material

*A. sativum* genotype L13 and *A. ampeloprasum* L. plants were collected from the Botanical Garden of Maria Curie-Skłodowska University in Lublin. *A. sativum* has been grown in the collection of the Botanical Garden since 1970 and propagated exclusively in the vegetative mode using daughter bulbs, the so-called cloves. In turn, *A. ampeloprasum* was propagated from seeds. The study material comprised *A. sativum* anthers sampled from the individual flowers in the inflorescence covered by spathe, which was removed manually. Meiosis occurred in flower buds, and no full anthesis was observed (absence of the anthesis stage). Since meiosis in *A. sativum* is asynchronous, all meiotic stages were found simultaneously in the sampled anthers. After meiosis in *A. sativum*, the single flowers did not develop but the sterile inflorescence elements were expanding. Anthers from single *A. ampeloprasum* flowers were collected from the inflorescences after spathe opening, from flowers before anthesis (for analyses of microsporogenesis) and after anthesis (for investigations of pollen viability and *in planta* germination). The material was sampled randomly from 50 plants throughout the period of microsporogenesis taking place in the anthers (ca. 1 month). Since there was no correlation between the length of the flower bud and the meiotic stage in the sporogenous tissue cells, selection of the material in both species was performed on the basis of analysis under a light microscope of crushed preparations of acetocarmine-stained anthers. Each microsporogenesis stage was subjected to analysis on minimum 30 meiotically dividing cells. Mitotically dividing somatic garlic cells originated from the apical meristematic part of *A. sativum* roots were used as a control for immunocytochemical staining.

### Phylogenetic analysis

Total DNA was isolated from leaves cut from *A.sativum* genotype L13 and *A. ampeloprasum* with the CTAB method (Doohan et al. 1998). The ITS (internal transcribed sequence of the ribosomal DNA–rDNA) region was amplified using ITSA and ITSB primers according to the procedure described previously by Shemesh Mayer et al. (2013). The 0.75-kb ITS amplicon purification steps and sequencing were carried out as described by Błaszczyk et al. (2011). Consensus sequences were identified by the BLAST program (Altschul et al. 1990). The comparative analysis was based on ITS sequences obtained in the present study for *A. ameloprasum* and *A. sativum* genotype L13 and six ITS sequences retrieved from GenBank database as the closest matches (99–100 % homology): KF317636 (*A. ameloprasum* [leek], EU626314 (*A. ameloprasum* [kurrat]), EU626310 (*A. ameloprasum* [GHG]), FJ664340 (*A. ameloprasum* [pearl onion]), FJ664319 (*A. ameloprsum* [bulbous leek]), and EU626375 (*A. sativum*). The sequences were aligned using CLUSTAL W (Thompson et al. 1994).

For phylogenetic analysis, the DNA sequences were initially aligned with CLUSTAL W (Thompson et al. 1994) and then rechecked and adjusted manually using MEGA4 software (Tamura et al. 2007). *Allium fistulosum* (FJ664288) and *A. cepa* (FJ664287) of the subgenus *Cepa* were chosen as outgroups. Phylogenetic relationships were reconstructed with the MEGA4 software using the maximum parsimony approach (Close-Neighbour-Interchange algorithm with search level 1, in which the initial trees were obtained with random addition of sequences—10 replicates) with a complete deletion option (i.e. all positions containing gaps and missing data were eliminated from the data set). All reconstructions were tested by bootstrapping with 1000 replicates.

### Immunofluorescence method

Pieces of *A. sativum* and *A. ampeloprasum* anthers were fixed for 24 h in 4 % paraformaldehyde and 0.25 % glutaraldehyde in MT stabilising buffer (MSB) (Baluska and Barlow 1993), pH 7.0 at room temperature. They were then rinsed in MSB buffer, dehydrated, embedded in polyethylene glycol, and sectioned according to the method of van Lammeren et al. (1985). 2-µm-thick sections were made on a rotation microtome MICROM HM340 and mounted on slides coated with 2 % organosilan (Sigma), and the slides were rinsed three times for 5 min each in phosphate-buffered saline (PBS). Next, they were treated with 0.1 M NH_4_Cl in PBS, washed twice for 5 min in PBS, and blocked with 0.1 % bovine serum albumin (BSA) in PBS for 30 min. Subsequently, the slides were incubated in a moist chamber for 60 min at 37 °C with monoclonal anti-mouse β-tubulin (Sigma) diluted 1: 200 in 0.1 % BSA in PBS. After washing with 0.1 % BSA in PBS (three times for 15 min), incubation with a secondary antibody was carried out for 60 min at 37 °C. The secondary antibody, conjugated with fluorescein isothiocyanate (Sigma), was diluted 1: 200 in PBS with 0.1 % BSA. 4′,6-diamidino-2-phenylindole dihydrochloride (DAPI) was added to the sections to stain DNA in the nuclei and organelles. In order to verify the immunocytochemical reaction, mitotically dividing somatic garlic cells were used after preparation with the procedure described above. Images of the sections were collected on a laser scanning confocal microscope LSM780 Zeiss with ZEN2010 data acquisition software using a Plant Apochromat 63x/1.40 Oil DIC M27 objective. Two-channel imaging was performed with excitation light set at 488 nm from an Argon laser for FITC and at 405 nm from a diode laser for DAPI. Fluorescence emission was recorded in the range of 500–560 and 410–460 nm, respectively. Both lasers worked at 2 % power to avoid photobleaching.

### Electron microscopy

For transmission electron microscopy (TEM), the *A. sativum* and *A. ampeloprasum* anthers were fixed in 2.5 % paraformaldehyde and 2.5 % glutaraldehyde in 0.1 M phosphate buffer (pH 6.9) for 24 h at room temperature. The specimens were washed three times in phosphate buffer and post-fixed in 2 % osmium tetroxide. Afterwards, they were dehydrated in a graded ethanol series and embedded in London Resin White Medium (Sigma). Ultrathin sections (60 nm) were stained with uranyl acetate (5 min) and lead citrate (10 min). The sections were observed under a JEM 100B transmission electron microscope.

### Pollen viability and in planta germination in* A. ampeloprasum*

*A. ampeloprasum* pollen grains were collected immediately after anthesis and stained with the Alexander assay according to the method of Peterson et al. (2010). To test *in planta* germination capacity, the pollen grains were placed on the stigma. The analyses were performed on 10 randomly chosen pistils in three replicates. After 24 h, styles were sampled and placed in a 0.1 % solution of aniline blue for 30 min. Next, the styles with stigmas were delicately crushed on glass slides, and germinating pollen grains were observed under a Nikon Eclipse N*i* fluorescence microscope using a 330- to 380-nm excitation filter and a 420-nm cut-off filter. In order to calculate the per cent of germinating pollen grains, pollen with pollen tubes penetrating stigma cells or style tissue were analysed under a light microscope. Photographic documentation was made with a digital camera and NIS-Elements BP software.

## Results

### Phylogenetic analysis

**Fig. 1:**
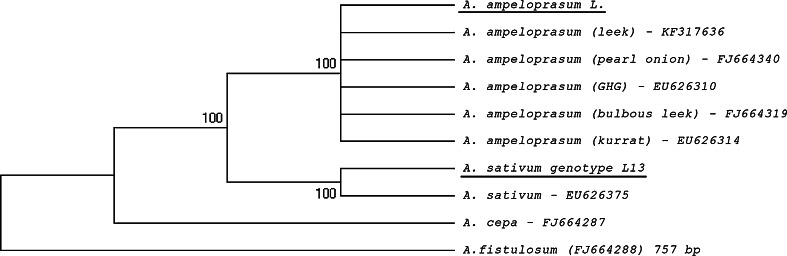
Consensus tree of maximum parsimony analyses based on the ITS sequences data set of different accession of *A. ameloprasum* and their closely related *A. sativum* species. The *underline* indicates sequences of *A. ameloprasum* L. and *A. sativum* L13 obtained in the present study. *Allium fistulosum* and *A. cepa* were used as an outgroup in the analysis. Bootstrap values higher than 50 % (1000 replicates) are shown at each branch

In order to estimate the relationships between the two analysed plants, *A. sativum* L13 and *A. ameloprasum* L., genetic comparative analysis was performed. The rDNA fragments from analysed species, which correspond to ITS (internal transcribed sequence), were cloned and sequenced. The DNA sequences in the analysed species were in a range between 714 bp (*A. ameloprasum* L.) and 745 bp (*A. sativum* L13). The analysis involved 10 nucleotide sequences, and there were 625 positions in total in the final data set, of which 497 were conserved, 126 were variable, and 105 were parsimony informative. Maximum parsimony (MP) analysis generated 83 equally parsimonious trees with a length of 131 steps and a consistency index CI = 0.992, retention index RI = 0.992, and rescaled consistency index RCI = 0.985. The results are presented as a consensus tree (Fig. 1). The phylogenetic analysis revealed two well-supported clades with strong bootstrap values of 100 %, clearly separated from the outgroup species of *A. fistulosum* and *A. cepa*. The first clade included a monophyletic group formed by different accessions of *A. ameloprasum*. The second clade represents the *A. sativum* species, which appears as a sister to the *A. ameloprasum* clade. Thus, this shows that the analysed *A. sativum* L13 and *A. ampeloprasum* L. belong to two different but closely related species.

### Morphological analysis

**Fig. 2:**
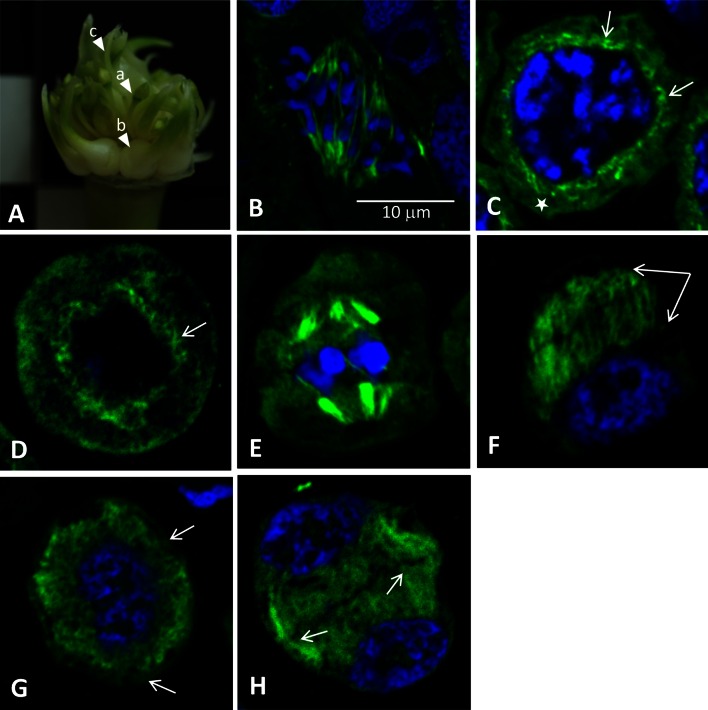
*A. sativum* L13: **A** inflorescence *a*-flower, *b*-topsets, *c*-leaf-like bracts. **B** Mitotically dividing meristematic root cell in early anaphase. MTs of the karyokinetic spindle visualised by indirect immunofluorescence (*green colour*). Chromosomes stained with DAPI (*blue colour*). **C**–**H** Microsporocytes of *A. sativum* during meiosis I. **C**, **D** Prophase I, **C** visible short fragments of MTs surrounding the cell nucleus (*arrows*) and parietal cytoplasm devoid of MTs (*star*), **D** tangential section—visible crossing MTs (*arrow*) around the nucleus, the absence of visible nuclear chromatin, as it is strongly condensed and distributed irregularly within the nucleus. **E** Metaphase I, visible karyokinetic spindle and metaphase chromosomes. **F**–**H** Telophase I, **F** tangential section—visible phragmoplast (*arrows*) and one of the two telophase nuclei, **G** diagonal section—visible one of the two telophase nuclei and radially arranged MTs of the phragmoplast, only a small fragment of the parietal cytoplasm was devoid of MTs (*arrows*). **H** An undulating primary septum visible between two nuclei and MTs in the parietal cytoplasm (*arrows*). Figures **B**–**H** are in the same magnification

The aim of the investigations was to compare the tubulin cytoskeleton during microsporogenesis in male-sterile garlic and fertile leek. The microsporogenesis process takes place in flowers; in *A. sativum* L13, the process occurred in pre-anthesis flowers. The green colour of the anthers in such flowers did not change throughout the microsporogenesis period. The inflorescences of the investigated *A. sativum* L13 have few flowers, bulbils, the so-called topsets, and transformed bracts in the form of elongated leaf-like bracts (Fig. 2A a–c). In turn, the inflorescence in *A. ampeloprasum* is composed of only fertile flowers without vegetative organs. In this species, a gradual change in the anther colour from green (pre-anthesis) to dark purple (anthesis) was observed throughout the microsporogenesis process (Fig. 4A).

### Immunofluorescence analysis

Verification of the immunocytochemical MT labelling method in the garlic microsporocytes involved visualisation of microtubules in the control material, i.e. in mitotically dividing somatic root cells. The immunocytochemical method applied facilitated unambiguous identification of microtubules in the dividing cells. Besides, the stage of dividing cells can be identified by the condensation of DAPI-stained DNA. In the analysed mitotically dividing cells in early anaphase, normal staining of karyokinetic spindle microtubules (green) and chromosomes (blue) was observed (Fig. 2B). In the next step, a subset of garlic and leek meiotic cells was observed. Garlic prophase meiocytes at the pachytene stage had a tubulin cytoskeleton in the form of short fragments surrounding the cell nucleus (Fig. 2C-arrows), while the other part of the cytoplasm was devoid of microtubules (Fig. 2C-star). In such cells, MTs formed a dense network of crossing fragments around the nucleus, which was clearly visible in the tangential sections of meiocytes (Fig. 2D-arrow). In turn, in prophase I meiocytes of the fertile leek, MTs formed a dense, well-organised network that was evenly distributed across the cytoplasm (Fig. 4B). As shown by the tangential section through a prophase meiocyte in the leek (Fig. 4C), the network was considerably denser than in the male-sterile garlic (Fig. 2D). During metaphase I in garlic meiocytes, all MTs present in the cytoplasm formed a karyokinetic spindle composed of few but thick MT filaments (Fig. 2E). In contrast, the karyokinetic spindle formed by MTs in the metaphase in leek contained more numerous and clearly thinner MT filaments, (Fig. 4D). In telophase I in garlic, a large phragmoplast appeared in the meiocyte equatorial plane between the daughter nuclei. The MTs of the phragmoplast formed dense, long filaments extending from the nuclei to the equatorial plane of the cell (Fig. 2F-arrows). The tangential section of one of the two daughter nuclei revealed radially arranged MTs of the phragmoplast. They occupied almost the entire volume of the cell, and only a small fragment of the parietal cytoplasm was devoid of MTs (Fig. 2G-arrows). The telophase meiocytes in leek exhibited a well-developed phragmoplast composed of numerous, regularly arranged MTs (Fig. 4E). In garlic and leek, successive cytokinesis occurs, during which a characteristic undulating primary septum is formed. In such cells in garlic, thick MT filaments were observed in the parietal cytoplasm, indicating centrifugal depolymerisation of the phragmoplast (Fig. 2H-arrows). Such depolymerisation of the phragmoplast took place in leek as well, but the MTs visible in the parietal cytoplasm in this species were composed of numerous, densely arranged MT filaments (Fig. 4F).

**Fig. 3:**
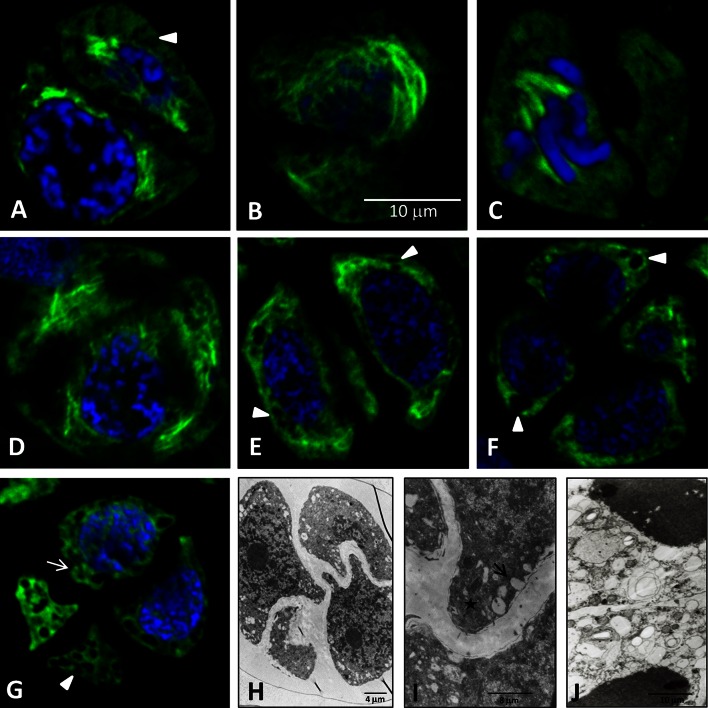
Microsporocytes of *A. sativum* during meiosis II. **A**–**G** MTs visualised by indirect immunofluorescence (*green colour*). Nuclear DNA stained with DAPI (*blue colour*). **H**, **I** TEM. **A**, **B** Prophase II, long and thick filaments of MTs surrounding the nucleus, *arrowheads*—*black* spots devoid of the cytoplasm and indicating cytoplasm decay. **B** Visible one of the two dyad cells. **C** Metaphase II—visible one of the two dyad cells with a karyokinetic spindle and metaphase chromosomes. **D** Telophase II, diagonal section—visible one of the four telophase nuclei and irregularly arranged filaments of the MTs forming the phragmoplast. **E**–**G** Tetrads of microspores, **E** visible two of the four tetrad cells with short fragments of MTs scattered in the cytoplasm. **G** Tubulin visible as single points dispersed in the cytoplasm—*arrow*. **E**–**G** *Arrowheads*—*black* spots devoid of the cytoplasm and indicating cytoplasm decay. Figures **A**–**G** are in the same magnification. **H**, **I** Tetrads of microspores visualised under TEM, **I** visibly lytic vacuoles (*arrow*) and electron-dense deposits (*star*). **J** Tetrads of *A. ampeloprasum,* visible two of the four microspores with normally developed cell organelles (TEM)

**Fig. 4:**
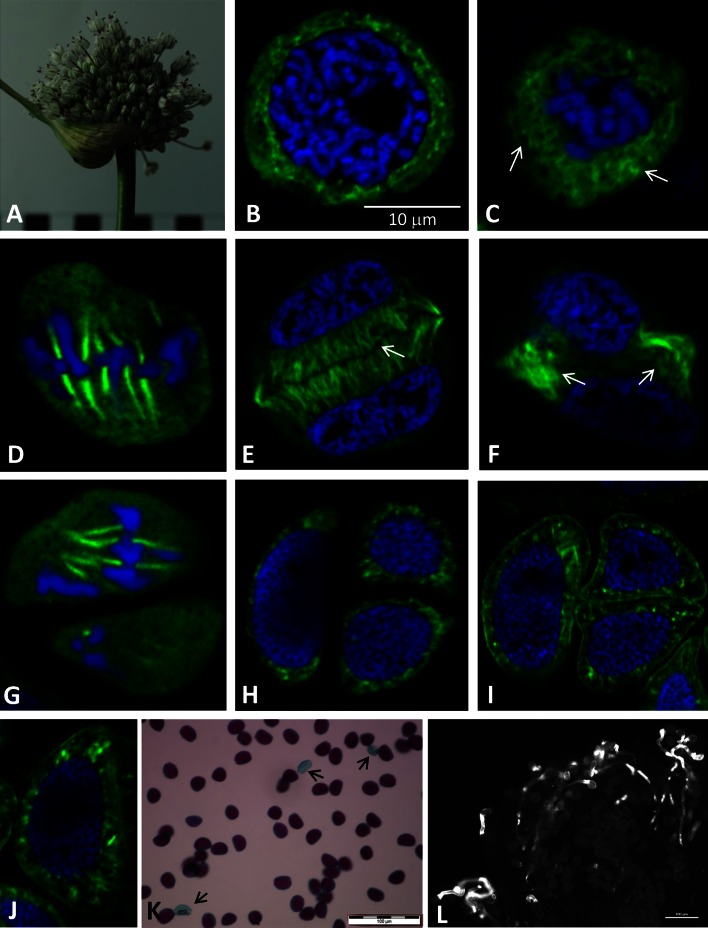
*A. ampeloprasum* L. **A** inflorescence **B**–**I** Microsporocytes during meiosis I and II. MTs visualised by indirect immunofluorescence (*green colour*). Nuclear DNA stained with DAPI (*blue colour*). **B**, **C** Prophase I, visible dense network that was evenly distributed across the cytoplasm (*arrows*). **D** Metaphase I, visible more numerous and clearly thinner MT filaments and metaphase chromosomes. **E**, **F** Telophase I, **E** a well-developed phragmoplast with a well-visible primary septum between two telophase nuclei (*arrow*), **F** visible MTs in the parietal cytoplasm (*arrows*). **G** Metaphase II, the *diagonal* section shows one of the two cells with the karyokinetic spindle and chromosomes forming a metaphase plate. **H**, **I** Tetrads of microspores with developing cortical MTs. **J** Single microspore with visible, well-developed cortical MTs arranged radially around the nucleus. Figures **B**–**J** are in the same magnification. **K** Pollen grains stained with the Alexander assay; viable grains stained purple, dead pollen grains stained *green* (*arrows*). **L** Germination of pollen grains on the stigma—visible fluorescence of the callose wall of the pollen tube stained with aniline *blue*

The second meiotic division, both in garlic and leek, proceeded within the microspore dyad. In prophase II garlic meiocytes, the phragmoplast disappeared and tubulin cytoskeleton filaments were visible only near the nucleus (Fig. 3A). The tangential section through one of the two dyad cells showed long and thick filaments of MTs surrounding the nucleus (Fig. 3B). During metaphase II, all MTs present in the cytoplasm formed a karyokinetic spindle composed of few thick MT filaments (Fig. 3C). In turn, metaphase II leek meiocytes exhibited MTs forming a karyokinetic spindle made up by long, thin filaments (Fig. 4G), which were more abundant than in garlic. In early telophase II in garlic, the long and irregularly arranged filaments of the tubulin cytoskeleton formed the phragmoplast, which was well visible in the diagonal section (Fig. 3D). The microspore tetrads were formed after the meiotic division and surrounded by a common callose wall, with the presence of short fragments of MTs scattered in the cytoplasm (Fig. 3E, F). In older tetrads, MTs were visible as single points, suggesting that MTs underwent gradual depolymerisation resulting in disappearance of tubulin filaments (Fig. 3G arrow). In the cytoplasm of such cells, there were many globular spaces devoid of the cytoplasm, which constituted a cytological image of degeneration thereof. In the course of meiotic division II, a gradual increase in the number of this type of spaces was observed (Fig. 3A–G arrowheads). In *A. ampeloprasum*, no such changes in the cytoplasm were observed at any of the microsporogenesis stages; in turn, MTs in the microspore tetrads formed a cortical cytoskeleton composed of fragments arranged radially around the nucleus (Fig. 4H). In post-meiotic cells in this species, the callose envelope was hydrolysed, and microspores were gradually released. Such microspores exhibited an extensive network of cortical MTs and a post-meiotic wall surrounding each cell (Fig. 4I, J).

### Electron microscopy analysis

The development of the male gametophyte in *A. sativum* L13 is inhibited at the stage of mononuclear microspores enveloped with a common callose wall. Such microspores observed under the electron microscope (TEM) were characterised by cytoplasm decay. Although a centrally located nucleus with a nucleolus was visible in the cells, there were no ER channels, protoplastids, or mitochondria in the cytoplasm. In turn, there were small lytic vacuoles scattered in the cytoplasm and electron-dense deposits, i.e. probably degenerated cell organelles (Fig. 3H, I). In the control material, i.e. *A. ampeloprasum* tetrads viewed under TEM, the cytoplasm contained normally developed cell organelles: the nucleus, mitochondria, plastids, and ER channels, which were distributed evenly in the cytoplasm (Fig. 3J).

### Pollen viability and germination analysis in *A. ampeloprasum*

After microsporogenesis of leek, there were viable pollen grains capable of germinating in the pollen tube. As shown by the Alexander assay, the pollen grain viability in the analysed leek was 85–90 %. In this assay, viable pollen grains stained purple (protoplast) and green (cell wall). Dead pollen grains stained only green (Fig. 4K-arrows). Additionally, in order to assess the ability of the pollen grains to germinate, *in planta* (on stigma) analyses were performed. Aniline blue staining showed the callose walls of the pollen tubes germinating on the stigma and penetrating the style. A germination rate of 30–40 % of the leek pollen grains was demonstrated (Fig. 4L).

## Discussion

*A. sativum* L13 is a sterile genotype with exclusively vegetative propagation, in which the development of the male gametophyte is inhibited at the stage of mononuclear microspores enveloped by a common callose wall (Winiarczyk et al. 2012; Shemesh Mayer et al. 2015). In contrast, *A. ampeloprasum*, belonging to the same genus, propagates through seeds (Jones and Mann 1963; Mathew 1996). Both species are economically important, and with this in mind, vegetative propagation is often preferred as it offers a possibility of conservation of features desired by growers. On the other hand, sexual reproduction promotes recombination of genes, which contributes to variability and plant adaptation to changing environmental conditions. Therefore, elucidation of the mechanisms of male sterility in garlic is essential, as it may be a basis for overcoming sterility in this important crop plant.

In order to cast more light on the male sterility of garlic, comparative analyses of two closely related species from the genus *Allium* were carried out. Initially, the investigations involved verification of the species used in the study by genetic analysis to establish the relationship between *A. sativum* L13 and *A. ampeloprasum.* The analysed ITS region of the rDNA gene cluster has been successfully used to assess the phylogenetic relationship among the species of the genus and subgenus *Allium* (Dubouzet and Shinoda 1998, 1999; Mes et al. 1999; Fritsch and Friesen 2002; Ricroch et al. 2005; Friesen et al. 2006; Gurushidze et al. 2007, 2008; Ipek et al. 2008; Nguyen et al. 2008; Li et al. 2010). The phylogenetic analysis led to differentiation of *A. ampeloprasum* L. and *A. sativum* into separate clusters. However, according to ITS nucleotide sequences, *A. sativum* genotype L13 was found to be more similar to the *A. ameloprasum* group than to *A. fistulosum* and *A. cepa*. The molecular evidence provided in this study is consistent with the reported observations of the genetic diversity and relationship within the section *Allium* (Figliuolo and Di Stefano 2007; Hirschegger et al. 2010; Guenaoui et al. 2013). Moreover, our results support the findings of Guenaoui et al. (2013), who conclude that the *A. ameloprasum* group can include or be very close to *A. sativum* when ITS sequence variation is used as a molecular marker; therefore, leek was used in this study as comparative material.

In *A. sativum* L13 investigated in this paper, the sterile elements of inflorescences are predominant; they press and crush each other, thereby posing a threat to the delicate flowers. Furthermore, the sterility in *A. sativum* L13 described earlier was classified as sterility type 1, which is defined as dysfunction of the development of both male and female gametophytes (Shemesh Mayer et al. 2013). Hence, the sterility in garlic genotype L13 may be associated with the inability to produce individual flowers and male and female sterility of differentiated individual flowers. In turn, a majority of fertile species from this genus have inflorescences devoid of vegetative organs (Keller 2002). The *A. ampeloprasum* inflorescence is composed of only flowers, which eliminates possible adverse factors limiting the development of normal flowers already at this stage. However, the changes observed in the inflorescence structure in garlic L13 are probably not the major cause of male sterility, since investigations involving removal of sterile elements (topsets) did not yield positive results, i.e. production of a large number of viable seeds (Konvicka 1984, own unpublished data).

Therefore, disturbances in the development of generative cells may be suggested as one of the main causes of male sterility in flowering plants. As shown in numerous reports, microtubules, i.e. the main component of the cytoskeleton in the plant cell, play an important role in the meiosis process (Brown and Lemmon 1988, 1991, 1996; Staiger and Cande 1990; Tchórzewska et al. 2008). So far, no information has been provided about the tubulin cytoskeleton during microsporogenesis in the male-sterile *A. sativum*. During microsporogenesis, reorganisation of the tubulin cytoskeleton begins already at the stage of early-prophase meiocytes and continues until the final changes in the telophase cell. The investigations presented in this paper show that during karyokinesis and cytokinesis in meiotic divisions I and II in garlic L13 and leek, similar in both species, the microtubular cytoskeleton formed a perinuclear network in prophase I and prophase II, a karyokinetic spindle in metaphase I and metaphase II, and a phragmoplast in telophase I and telophase II. These three basic configurations are typical for angiosperms (Hogan 1987; Traas et al. 1989; Brown and Lemmon 1996, 2000). Similar observations were described in *Triticum aestivum* stressed with low temperature during microsporogenesis. The stress did not induce changes in MT configurations in the consecutive meiosis stages; nevertheless, there were disturbances in the functional development of pollen grains (Barton et al. 2014). In the present paper, it was observed that the tubulin cytoskeleton in garlic L13, despite formation of the three basic configurations, was distinctly poorer (composed of a few MT filaments) compared with that in the meiotically dividing cells in *A. ampeloprasum* (see Figs. 2D–4C, 2E–4D, 2H–4F, 3EFG–4HIJ). Remarkably fewer MT filaments formed the cytoskeleton in the garlic microsporocytes in prophase I and metaphase I as well as the dyad stage, when depolymerisation of MTs took place. Also during meiotic division II, the tubulin cytoskeleton was composed of a few thick MT filaments, despite the normal configurations. In turn, *A. ampeloprasum* meiocytes at all microsporogenesis stages had a rich, well-developed tubulin cytoskeleton composed of numerous thin MT filaments. As in the leek, a well-developed tubulin cytoskeleton in meiotically dividing cells was reported for *Allium cepa* (Zhang et al. 2011), which is a fertile species related to garlic and leek, as shown by the phylogenetic analysis.

The tubulin cytoskeleton not only plays a direct role in meiotic cell division but also serves an important function in intracellular transport and, hence, in the control of plant morphogenesis (Green 1962). For instance, the cortical MTs control the direction of cell expansion by orienting microfibril deposition in the cell wall (Giddings and Staehelin 1991; Shibaoka 1994; Cyr and Palevitz 1995; Bulbert et al. 1998; Hasezava and Nozaki 1999). Therefore, in this study, attention was paid to the tubulin cytoskeleton in post-meiotic cells, i.e. microspores. In the garlic microspore tetrads, the tubulin cytoskeleton not only was clearly poorer than in the leek but also underwent gradual depolymerisation. In older garlic tetrads, only point tubulin fluorescence was observed, whereas the leek microspores had an extensive network of cortical MTs. Furthermore, degenerative changes in the cytoplasm of garlic microspores, i.e. a sign of the process of cell dying that prevents the onset of gametogenesis, were observed. The close correlation between the microsporogenesis course, formation of functional pollen grains, and the tubulin cytoskeleton has also been described in *Lavatera thuringiaca*, in which the microsporogenesis process was disturbed by treatment of meiocytes with colchicine—an inhibitor of MT lengthening. The drastic action of colchicine inhibited meiosis at each stage of microsporogenesis (Tchórzewska et al. 2008).

In summary, the presented results show that, during microsporogenesis, the tubulin cytoskeleton in male-sterile garlic *A. sativum* L13 formed normal configurations on the one hand but was considerably poorer than that in the fertile *A. ampeloprasum* on the other. These differences did not affect the course of karyokinesis, chondriokinesis, and cytokinesis, which contributed to completion of microsporogenesis. In turn, at the onset of the successive stage of the development of fertile pollen grains, i.e. gametogenesis, the disturbances observed involved the absence of a normal cortical cytoskeleton and dramatic cytoplasm degeneration. It seems that, due to disturbances in cortical cytoskeleton formation at the very beginning of gametogenesis, the intracellular transport governed by the cytoskeleton might be perturbed, leading to microspore decay in *A. sativum* L13. In the future, it would be interesting to compare the behaviour of MTs during microsporogenesis and gametogenesis of male-sterile *A. sativum* L13 and fertile garlic phenotypes, whose natural habitats are mainly located in inaccessible regions of Central Asia (Etoh et al. 1988; Pooler and Simon 1993).

### **Author contribution statement**

DT conceived study, designed, performed experiments, analyzed, interpreted data, wrote the manuscript; KD contributed to Figs. 3, 4 and 5; LB contributed to Figs. 1 and 2; KW conceived study, interpreted data.
